# The complete mitochondrial genome of *Thryssa setirostris* (Engraulinae, Engranlidae, Clupeoidei) and phylogenetic studies of Clupeoidei

**DOI:** 10.1080/23802359.2018.1437807

**Published:** 2018-03-09

**Authors:** Wei Chen, Hui Jiang, Xun Du, Li Gong, Liqing Liu, Bingjian Liu, Zhenming Lü

**Affiliations:** National Engineering Laboratory of Marine Germplasm Resources Exploration and Utilization, College of Marine Science and Technology, Zhejiang Ocean University, Zhoushan, People’s Republic of China

**Keywords:** *Thryssa setirostris*, mitogenome, phylogenetic relationship

## Abstract

The complete mitochondrial genome of *Thryssa setirostris* has been determined. The whole sequence was 16,926 bp in length and included 13 protein-coding genes, 22 transfer RNA genes, 2 ribosomal RNA genes, and 1 control region (D-loop). The overall base composition is A 29.88%, C 29.23%, G 16.77%, T 24.02%, with a slightly A + T bias of 54%. With the exception of *ND6* and eight tRNA genes, all other mitochondrial genes are encoded on the heavy strand. Phylogenetic tree was constructed based on 13 protein-coding genes sequences of 21 clupeoidei species, and the result showed that *T. setirostris* is most closely related to *T. dussumieri.* These mitogenome sequence data will be useful for population genetics and phylogenetic analysis of the Clupeoidei.

*Thryssa setirostris,* which belongs to Engraulinae, Engranlidae, Clupeoidei, is distributed in the western Indo-Pacific (Whitehead et al. 1988). So far, the complete mitochondrial genome (mitogenome) sequence of this species has not been reported yet. In order to find new DNA markers for the future research of population genetics and phylogenetics and taxology, we determined the complete mitogenome of *T. setirostris* (GenBank accession number MG755269) by PCR amplification and primer walking sequence method.

*Thryssa setirostris* was collected from the South China Sea (18°12′26″N 109°38′20″E) and stored in a refrigerator of −80 °C with accession number 20171209CHLT. The specimen was identified based on the morphologic features and *COI* gene. Muscle tissues of individual specimens for molecular analysis were reserved in ethanol absolute. Whole genomic DNA was extracted by using the phenol-chloroform method (Barnett and Larson 2012). Universal primers (Ivanova et al. 2007) were designed from the conserved regions of the complete mitochondrial genome sequences of 28 Clupeiformes species from GenBank database. Sequence alignment was conducted by BioEdit (Hall 1999). The phylogenetic tree involving 21 Clupeoidei species was constructed using the neighbour joining (NJ) methods based on the 13 protein-coding genes. The NJ trees were obtained with 10,000 bootstrap replications using MEGA5 (Tamura et al. 2011).

The complete mitochondrial genome of *T. setirostris* was 16,926 bp in length, consisting of 13 protein-coding genes, 22 transfer RNA genes (tRNA), 2 ribosomal RNA genes (12S rRNA and 16S rRNA), and 1 control region (D-loop). Except *ND6* and eight tRNAs (Gln, Ala, Asn, Cys, Tyr, Ser, Glu, Pro), other genes were encoded on the heavy strand. The mitochondrial base composition is A 29.88%, C 29.23%, G 16.77%, T 24.02%, respectively. The A + T content (54%) is higher than G + C content, in common with other Clupeoidei mitogenomes (Li et al. 2012; Bo et al. 2013; Zhang et al. 2016). Twelve protein-coding genes start with ATG except *COX1*, which start with GTG. For the stop codon, *ND6* ends with AGG, seven genes (*ND1*, *COX1*, *ATP8*, *ATP6*, *COX3*, *ND4L*, *ND5*) end with TAA, *ND2*, *ND3*, *COX2*, *ND4*, and *CYTB* with an incomplete TA or T. The 12S rRNA (952 bp) is located between tRNA^Phe^ and tRNA^Val^ genes, and 16S rRNA (1587 bp) is located between tRNA^Val^ and tRNA^Leu^ genes. The control region (D-Loop) typically located between tRNA^Pro^ and tRNA^Phe^ genes, is 1282 bp in length. Two tandem repeat sequences were observed in the control region (D-Loop). The shortest motif was 39 bp with five repeats, and the longest motif were 50 bp with two repeats. Such a symbolic structure of the control region are also frequently observed in other teleost fishes (Guo et al. 2003; Gong et al. 2015).

The phylogenetic tree (Figure 1) was constructed using the neighbour joining (NJ) method based on the 13 protein-coding genes of 21 clupeoidei species. The result shows that *T. setirostris* is most closely related to *T. dussumieri*. We expect the present results will further facilitate for the study on the taxonomy, population genetic structure, and phylogenetic relationships of Clupeoidei.

**Figure 1.**
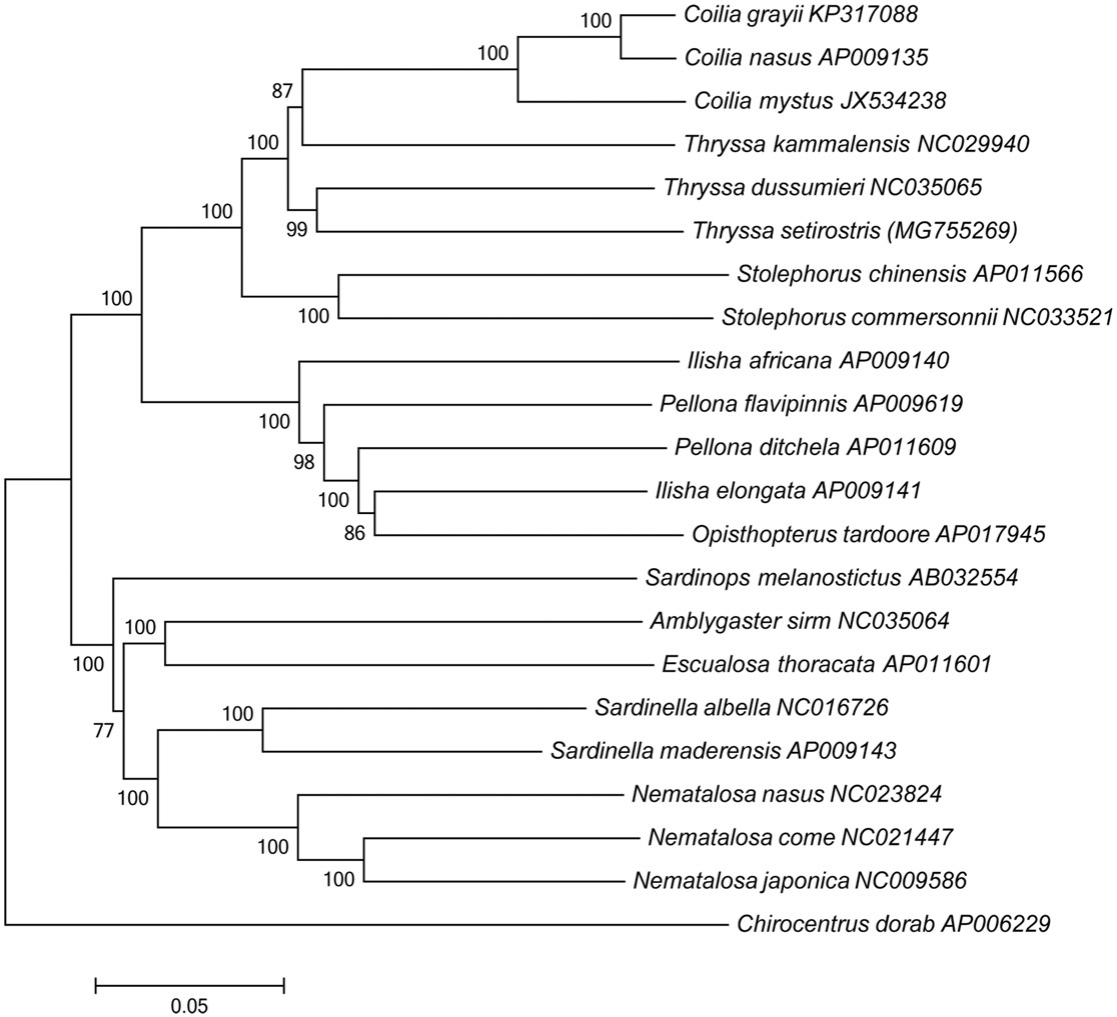
Neighbour-Joining tree was constructed based on 13 protein-coding genes of 21 Clupeoidei complete mitogenome. The black dot indicated the species in this study. The number at each node is the bootstrap probability. The number before the species name is the GenBank accession number.
